# Effect of group antenatal care versus individualized antenatal care on birth preparedness and complication readiness: a cluster randomized controlled study among pregnant women in Eastern Region of Ghana

**DOI:** 10.1186/s12884-024-06743-1

**Published:** 2024-08-16

**Authors:** Vida A. Kukula, Elizabeth Awini, Bidisha Ghosh, Veronica Apetorgbor, Ruth Zielinski, Georgina Amankwah, Winfred K. Ofosu, Katherine James, John E. O. Williams, Jody R. Lori, Cheryl A. Moyer

**Affiliations:** 1 Dodowa Health Research Center, Ghana Health Service, Dodowa, Ghana; 2 University of Michigan School of Nursing, Ann Arbor, MI USA; 3 Regional Health Directorate, Ghana Health Service, Koforidua, Eastern Region Ghana; 4 University of Michigan Medical School, Ann Arbor, MI USA

**Keywords:** Antenatal care, Group antenatal care, Prenatal care, Ghana, Sub-saharan Africa, Care seeking, Maternal morbidity, Neonatal morbidity, Maternal outcomes, Neonatal outcomes

## Abstract

**Background:**

As utilization of individual antenatal care (I-ANC) has increased throughout sub-Saharan Africa, questions have arisen about whether individual versus group-based care might yield better outcomes. We implemented a trial of group-based antenatal care (G-ANC) to determine its impact on birth preparedness and complication readiness (BPCR) among pregnant women in Ghana.

**Methods:**

We conducted a cluster randomized controlled trial comparing G-ANC to routine antenatal care in 14 health facilities in the Eastern Region of Ghana. We recruited women in their first trimester to participate in eight two-hour interactive group sessions throughout their pregnancies. Meetings were facilitated by midwives trained in G-ANC methods, and clinical assessments were conducted in addition to group discussions and activities. Data were collected at five timepoints, and results are presented comparing baseline (T0) to 34 weeks’ gestation to 3 weeks post-delivery (T1) for danger sign recognition, an 11-point additive scale of BPCR, as well as individual items comprising the scale.

**Results:**

1285 participants completed T0 and T1 assessments (*N* = 668 I-ANC, *N* = 617, G-ANC). At T1, G-ANC participants were able to identify significantly more pregnancy danger signs than I-ANC participants (mean increase from 1.8 to 3.4 in G-ANC vs. 1.7 to 2.2 in I-ANC, *p* < 0.0001). Overall BPCR scores were significantly greater in the G-ANC group than the I-ANC group. The elements of BPCR that showed the greatest increases included arranging for emergency transport (I-ANC increased from 1.5 to 11.5% vs. G-ANC increasing from 2 to 41% (*p* < 0.0001)) and saving money for transportation (19–32% in the I-ANC group vs. 19–73% in the G-ANC group (*p* < 0.0001)). Identifying someone to accompany the woman to the facility rose from 1 to 3% in the I-ANC group vs. 2–20% in the G-ANC group (*p* < 0.001).

**Conclusions:**

G-ANC significantly increased BPCR among women in rural Eastern Region of Ghana when compared to routine antenatal care. Given the success of this intervention, future efforts that prioritize the implementation of G-ANC are warranted.

**Trial registration:**

ClinicalTrials.gov Identifier: NCT04033003 (25/07/2019).

**Protocol available:**

Protocol Available at: https://www.ncbi.nlm.nih.gov/pmc/articles/PMC9508671/.

**Supplementary Information:**

The online version contains supplementary material available at 10.1186/s12884-024-06743-1.

## Background

Limited birth preparedness and complication readiness (BPCR) has been identified as a contributing factor to the persistent problem of high maternal and neonatal morbidity and mortality in low and middle income countries (LMICs) [1]. In Ghana, the maternal mortality ratio was estimated at 263 per 100,000 live births in 2020 [2]. BPCR is pivotal for maximizing maternal and newborn well-being [3, 4]. BPCR is based on the premise that being ready for potential complications reduces the first of Thaddeus and Maine’s Three Delays: delays in the decision to seek care [5]. (The other delays include getting to an appropriate obstetric facility and getting appropriate care once at the facility [5]. ) Since the decision to seek care is a necessary prerequisite to obtaining timely care, BPCR is an important catalyst for ensuring women get the care they need during birth and in the face of complications. [6] BPCR includes such behaviors as raising awareness of danger signs, improving problem recognition and reducing delay in deciding to seek care; choosing a birth location and provider in advance; knowing the location of the nearest skilled provider; obtaining basic safe birth supplies; and identifying someone to accompany the woman to the facility when labor begins [6–9]. It also includes setting aside money for transportation, identifying a blood donor, and having a plan for temporary family care in case of emergencies [6–9]. A meta analysis conducted by Soubeiga et al. [8] found implementation of BPCR interventions in low resource settings improved maternal and neonatal health and called for randomized trials to strengthen the data. BPCR is most often emphasized during routine antenatal care (ANC) visits. According to the 2022 Demographic Health Survey in Ghana, 88% of women had attended at least 4 ANC visits (in keeping with previous WHO recommendations) and approximately 40% of women had attended the most recent WHO recommendation of 8 + ANC visits [10, 11]. Nonetheless, another recent study in Ghana showed only 15% of women could spontaneously identify 3 out of the five basic 5 components of birth preparedness and complication readiness (which in that study was defined as 1) identified health facility for place of delivery, 2) saved money for the purpose of pregnancy and childbirth, 3) decided to deliver by skilled provider, 4) made advance arrangement for transport to skilled care health facility in case of emergency and 5) had made provision for delivery kit/materials). [12].

Knowing that antenatal care is where most pregnant women learn about how to be best prepared for delivery, we sought to explore whether group antenatal care (G-ANC) would yield improved birth preparedness and recognition of danger signs when compared to routine, individual antenatal care(I-ANC). The G-ANC model used is described in detail elsewhere [13]. In summary, women presenting for ANC are grouped by gestational age. A standard history and physical exam are completed during the initial visit, and group visits start at the second ANC visit. Prior to the start of each group, clinical assessments are conducted individually, and then the midwife and women sit in circle facing one another for a 60–90 min facilitated discussion. G-ANC uses story-telling, peer support, demonstration and teach-back to enhance its effectiveness [13].

A pilot study in Ghana by members of our team found G-ANC improved women’s health literacy, antenatal care access, birth preparedness, and postpartum planning [14]. Building on this work, we sought to rigorously test the differences in BPCR using a cluster randomized controlled trial methodology. The purpose of this paper is to quantify the differences in recognition of pregnancy danger signs, how to prevent problems, and BPCR measured at two time points between women randomized to G-ANC or I-ANC. Given routinely low levels of BPCR among women attending routine antenatal care, understanding the impact of G-ANC on BPCR is particularly critical. If G-ANC is able to significantly increase BPCR over standard ANC, it will provide evidence for one strategy to improve women’s preparation for birth and any potential complications.

## Methods

We conducted a five-year, cluster randomized controlled trial (cRCT) among 14 health facilities in the Eastern Region of Ghana, the details of which have been published elsewhere [13]. (ClinicalTrials.gov Identifier: NCT04033003 (25/07/2019), available at: DOI: 10.2196/40828; PMID: *36083608*) Health facilities within the region that had a sufficiently large volume of ANC registrants per month (at least 10 per month to allow for the formation of a group large enough for G-ANC) and whose new ANC registrants included women in the first trimester were selected for participation. Facilities were grouped into pairs with similar characteristics (facility type, district, number of monthly ANC registrants) and then randomized to G-ANC (intervention) or individual ANC (I-ANC) (control).

### Study setting

This study took place in four districts (Akwapim North, Yilo Krobo, Nsawam-Adoagyiri and Lower Manya Krobo) within the Eastern Region of Ghana between July 2019 and July 2023. Ghana is divided into 16 administrative regions, with the Eastern Region situated north and adjacent to the region that includes the capital city of Accra, the Greater Accra Region. Subsistence and commercial farming are anchors of the economy in the Eastern Region, and 25% of residents report having a primary school education or less [10]. The fertility rate for the region is similar to the national average (3.8 vs. 3.9) [10].

### Sampling and randomization

The number of monthly deliveries and the average gestational age of women presenting for ANC in each facility was used as the criteria for matching facilities, so that each pair of facilities were similar to each other with regard to these matching factors. One facility within each pair was randomly assigned to G-ANC (intervention), and the other was assigned to I-ANC (control). Details of the randomization procedure and sample size calculations have been described elsewhere [13].

### Recruitment

Women were recruited at each of the 14 participating health facilities. Clinic staff assisted a trained Research Assistant (RA) in identifying women who might be eligible to participate. Inclusion criteria stipulated that women must be pregnant and (1) less than 20 weeks gestation; (2) able to speak Dangme, Ga, Akan, Ewe, or English; (3) older than 15 years of age; and (4) not be considered high risk. Trained RAs talked to women about the study, and those who were willing to participate were taken through an informed consent procedure and completed baseline data collection.

### Data collection

REDCap, a secure web application, was used to capture all quantitative data. Trained RAs administered all surveys using a password protected tablet that could be used with or without wifi, allowing them to store data on the tablet if it was necessary to work offline. Data collection occurred at five time points: T0: baseline; T1: 34 weeks’ gestation to 3 weeks postdelivery; T2: 6–12 weeks after delivery; T3: 5–8 months postpartum; and T4: 11–14 months postpartum. Data presented here focus on T0 and T1; future analyses will explore longer term impact of G-ANC using post-delivery data. Data presented here include demographics, health history, pregnancy history, and questions related to birth preparedness and complication readiness and were collected at baseline (T0) and 34 weeks’ gestation to 3 weeks post-delivery (T1).

Knowledge of Danger Signs During Pregnancy (DSDP) included 14 items that women were asked to recall, unprompted, including: headache; vision changes / blurred vision; pain in abdomen; shortness of breath; fever; vaginal bleeding; leaking of fluids from vagina; painful urination; signs of labor before it is time; reduced or no fetal movement; convulsions or fits; persistent vomiting; mood changes; swollen face. DSDP items were examined individually as well as aggregated into a 14-point scale. Only those items with enough responses to analyze are reported in Table 1. (Shortness of breath, painful urination, reduced or no fetal movement, and mood changes were not able to be modeled.)

**Table 1:** Participant demographics

Categorical Variables *n* (%)		Overall *N* (Column percents)	I-ANC *N* (Row percents)	G-ANC *N* (Row percents)	*P*-value
		*N* = 1761*	*N* = 884	*N* = 877	
Age Category					
	Less than 25	501 (28.4)	266 (53.1)	235 (46.9)	0.193
	25–34	987 (56.0)	477 (48.3)	510 (51.7)	
	35 or more	273 (15.5)	141 (51.6)	132 (48.4)	
Maternal Education (*N* = 1698)					
	Primary	246 (14.5)	120 (48.7)	126 (51.2)	0.690
	Middle/JHS/JSS	829 (48.8)	429 (51.7)	400 (48.3)	
	Secondary/SHS/Technical/Vocational	459 (27.0)	223 (48.6)	236 (51.4)	
	Tertiary	164 (9.7)	83 (50.6)	81 (49.4)	
Partner Education (*N* = 1691)					
	Middle/JHS/JSS or less	666 (39.4)	335 (50.3)	331 (49.7)	0.853
	Secondary	627 (37.1)	306 (48.8)	321 (51.2)	
	Tertiary	261 (15.4)	126 (48.3)	135 (51.9)	
	N/A, Unknown	137 (8.1)	64 (46.7)	73 (53.3)	
Religion					
	Christianity	1646 (93.5)	835 (50.7)	811 (49.3)	0.119
	Muslim	97 (5.5)	39 (40.2)	58 (59.8)	
	Other	18 (1.0)	10 (55.6)	8 (44.4)	
First pregnancy					
	No	1412 (80.2)	703 (49.8)	709 (50.2)	0.488
	Yes	349 (19.8)	181 (51.9)	168 (48.1)	
Place of Delivery					
	Hospital/Polyclinic/Health Center	1711 (97.2)	853 (49.9)	858 (50.1)	0.090
	Other	50 (2.8)	31 (62.0)	19 (38.0)	
Continuous Items: Mean (SD)					
Maternal age		28.2 (5.8)	28.1 (6)	28.3 (5.6)	0.504
Wealth index		6.8 (2.4)	6.9 (2.4)	6.9 (2.3)	0.617
Number of previous pregnancies		3.5 (1.4)	3.5 (1.4)	3.5 (1.5)	0.708

* Total sample size. Missing data are reflected in reduced sample sizes indicated next to variable names

Categorical variables were compared using chi-square test

Maternal age and wealth index tested using 2-sample t-test

Number of previous pregnancies tested using Mann Whitney Wilcoxon test (non-parametric)

Preparation for Birth (PB) included 11 items that women were asked to recall, unprompted. These included arranging for emergency transport, saving money, having a valid National Health Insurance card, obtaining supplies for the birth, keeping self clean, eating and drinking light food, watching out for health problems, identifying a place for delivery, identifying a blood donor, identifying someone to go with the woman to the facility for birth, and identifying someone else to care for other family members. PB items were examined individually as well as aggregated into an 11-point scale. Only those items with enough responses to analyze are reported in Table 2. (Having a valid National Health Insurance card and identifying a blood donor were not able to be modeled.)

**Table 2:** Recognition of danger signs during pregnancy

Danger Signs During Pregnancy items	Time = 0				Time = 1				*P*-values
	I-ANC (*N* = 884)		G-ANC (*N* = 877)		I-ANC (*N* = 668)		G-ANC (*N* = 617)		
	%	95% CI	%	95% CI	%	95% CI	%	95% CI	
Headache	29.4	26.4,32.4	25.5	22.7, 28.4	44.6	40.8, 48.4	51.1	47.1, 55.0	0.002
Vision changes/blurred vision)	8.3	6.4, 10.1	6.3	4.7, 7.9	7.6	5.6, 9.6	12.5	9.9, 15.1	0.001
Pain in abdomen	36.2	33.0, 39.4	40.6	37.3, 43.8	47.8	44.0, 51.5	44.6	40.6, 48.5	0.028
Fever	15.1	12.7, 17.4	13.6	11.3, 15.8	23.4	20.1, 26.6	24.1	20.8, 27.5	0.364
Vaginal bleeding	31.5	28.4, 34.5	40.3	37.0, 43.5	40	36.3, 43.7	76.7	73.3, 80.0	< 0.001
Leaking of fluids from vagina	7.4	5.6, 9.1	8.1	6.2, 9.9	10.3	8.0, 12.6	44.3	40.3, 48.1	< 0.001
Signs of labor before time for baby to come	2	1.1, 3.0	2.7	1.7, 3.8	4.5	2.9, 6.1	11.2	8.7, 13.7	0.060
Convulsions/Fits	1.0	0.3, 1.6	0.2	0, 0.5	2.0	0, 3.0	7.8	5.6, 9.9	< 0.001
Persistent vomiting	32.4	29.2, 35.4	31.1	28.1, 34.2	25.6	22.2, 28.9	32.7	29.0, 36.4	0.007
Swollen face	1.7	0.8, 2.5	1.1	0.4, 1.8	1.2	0.3, 2.0	7.9	5.8, 10.1	< 0.001
	**Mean (SE)**	**95% CI**	**Mean (SE)**	**95% CI**	**Mean (SE)**	**95% CI**	**Mean (SE)**	**95% CI**	
14 point DSDP Scale	1.7 (0.5)	1.6, 1.8	1.8 (0.04)	1.7, 1.9	2.2 (0.05)	2.1, 2.3	3.4 (0.06)	3.3, 3.5	< 0.001

For all the DSDP items, estimated percentage, 95% CI and *p*-values reported from logistic models

For DSDP scale, mean(SE), 95% CI and *p*-values reported from poisson regression model

Knowledge of how to prevent problems before birth (PPB) included 11 items that women were asked to spontaneously recall. These included sleeping under an insecticide treated bednet, taking malaria prophylaxis, eating frequent balanced meals, drinking plenty of water, taking iron pills, taking folic acid tablets, practicing safe sex / using a condom, getting tested for HIV, getting exercise, avoiding tobacco, and avoiding alcohol. PPB items were examined individually as well as aggregated into an 11-point scale. Only those items with enough responses to analyze are reported in Table 3. (Practicing safe sex/ wearing a condom, getting tested for HIV, and avoiding tobacco were not able to be modeled.)

**Table 3:** Preparation for birth

Preparation for Birth (PB) items	Time = 0				Time = 1				*P*-values
	I-ANC (*N* = 884)		G-ANC (*N* = 877)		I-ANC (*N* = 668)		G-ANC (*N* = 617)		
	%	95% CI	%	95% CI	%	95% CI	%	95% CI	
Arrange for emergency transport	1.5	0.7, 2.2	2.2	1.2, 3.1	11.5	9.1, 13.9	41	37.1, 44.9	< 0.001
Save money	19.2	16.6, 21.8	18.6	16.0, 21.2	32	28.5, 35.6	72.9	69.4, 76.4	< 0.001
Obtain supplies for the birth / prepare birth bed	91	89.1, 92.8	91.2	89.3, 93.1	94.6	22.9, 96.3	96.1	94.6, 97.6	0.332
Keep self clean (bathing)	4.1	2.7, 5.4	4	2.7, 5.3	3.7	2.3, 5.2	7.1	5.1, 9.2	0.037
Eating and drinking light food	9.4	7.5, 11.3	7.5	5.8, 9.3	5.7	3.9, 7.4	11.5	8.9, 14.0	< 0.001
Watch out for health problems	9.4	7.5, 11.3	7.8	5.9, 9.5	10.2	7.9, 12.5	13.1	10.5, 15.8	0.031
Identify place for delivery	4.1	2.8, 5.4	3.3	2.1, 4.5	5.1	3.4, 6.8	15.1	12.2, 17.9	< 0.001
Identify someone to go with woman to facility	1.2	0.5, 2.0	2.3	1.3, 3.3	3.1	1.8, 4.5	20.3	17.1, 23.4	0.001
Identify someone to care for other family members	0.8	0.2, 1.4	0.9	0.3, 1.5	1.4	0.5, 2.2	10.2	7.8, 12.6	0.002
	**Mean (SE)**	**95% CI**	**Mean (SE)**	**95% CI**	**Mean (SE)**	**95% CI**	**Mean (SE)**	**95% CI**	
Prepare for birth 11 point scale	1.4(0.03)	1.4, 1.5	1.4 (0.03)	1.3, 1.5	1.7 (0.04)	1.6, 1.8	3.0 (0.1)	2.8, 3.1	< 0.001

For all the PB items, estimated percentage, 95% CI and *p*-values reported from logistic models

For PB scale, mean(SE), 95% CI and *p*-values reported from poisson regression model

### Data analysis

Data management, including computing summary statistics was done using SAS 9.4 (SAS Institute, Cary, NC), while data analyses were conducted using Stata 18.0 (StataCorp LLC, College Station, TX). Baseline data were used to compute the demographics summary in Table 4. Results from T0 and T1 were compared between the two study arms to assess the efficacy of the intervention over time. Logistic regression adjusted for clustering was used to analyze changes in the yes/no items corresponding to danger signs during pregnancy, preparing for birth and preventing problems before baby is born. For the scale items, Poisson regression (for the 14-point danger signs during pregnancy scale and 11-point preparing for birth scale) and negative binomial regression for the 11-point prevention of problems before baby is born scale, adjusted for clustering by participant ID was used to analyze the effects of intervention over time. For each of these models, the predictors/covariates considered were the study arm (G-ANC vs. I-ANC), time (T0 vs. T1) and the interaction effect between them. The interaction effect compares the two study arms by comparing their change in scores from T0 to T1. A *p*-value of < 0.05 for the interaction effect was considered significant. The *p*-values reported in tables are the omnibus *p*-values of the interaction effect from the models, since we are interested in studying and comparing the trend from T0 to T1 between the two study arms.

**Table 4:** Knowledge of how to prevent problems before birth

Preventing problems before birth (PPB)	Time = 0					Time = 1				*P*-values
	I-ANC (*N* = 884)			G-ANC (*N* = 877)		I-ANC (*N* = 668)		G-ANC (*N* = 617)		
	%	95% CI	%		95% CI	%	95% CI	%	95% CI	
Sleep under insecticide treated bednet	14.7	12.4, 17.0	14.7		12.4, 17.1	15.1	12.4, 17.8	25.9	22.5, 29.4	< 0.001
Take malaria prophylaxis	3.4	2.2, 4.6	2.9		1.7, 4.0	6.6	4.7, 8.5	11.5	9.0, 14.0	0.016
Eat frequent balanced meals	64.7	61.6, 67.9	56.3		53.0, 59.6	78.6	75.5, 81.7	71.0	67.4, 74.6	0.717
Drink plenty of water	22.7	20.0, 25.5	21.7		18.9, 24.4	31.0	27.5, 34.5	34.5	30.8, 38.3	0.103
Take iron pills	25.2	22.4, 28.1	13.9		11.6, 16.2	32.9	29.4, 36.5	23.5	20.2, 26.8	0.102
Take folic acid tablets	27.2	24.2, 30.1	16.5		14.1, 19.0	33.4	29.8, 37.0	25.6	22.2, 29.1	0.105
Get exercise	10.8	8.7, 12.8	11.2		9.1, 13.3	17.1	14.2, 19.9	21.9	18.6, 25.1	0.186
Avoid alcohol	1.5	0.7, 2.3	4.2		2.9, 5.5	2.7	1.5, 3.9	5.7	3.8, 7.5	0.486
	**Mean (SE)**	**Mean (SE)**	**Mean (SE)**		**Mean (SE)**	**Mean (SE)**	**Mean (SE)**	**Mean (SE)**	**Mean (SE)**	
Prevent problem before birth 11 point scale	1.7 (0.1)	1.6, 1.8	1.4 (0.1)		1.4, 1.5	2.2 (0.1)	2.1, 2.3	2.3 (0.1)	2.1, 2.4	< 0.001

For all the PPB items, estimated percentage, 95% CI and *p*-values reported from logistic models

For PPB scale, mean(SE), 95% CI and *p*-values reported from negative binomial regression model

### Ethical approval

In accordance with the 1964 Declaration of Helsinki, the study protocol was reviewed and approved by the University of Michigan Health Sciences Behavioral Sciences Institutional Review Board (HUM00161464) and Ghana Health Service Ethics Review Committee (GHS-ERC:016/04/19). This study is registered on ClinicalTrials.gov, NCT04033003. All study participants were taken through and completed a written informed consent document. No participants were under the age of 16 and thus consent was obtained directly from each participant.

## Results

Table 1 illustrates the demographic characteristics of our sample, indicating that the intervention and control groups were well-balanced across key variables. One thousand seven hundred sixty one participants were recruited into the study, with 877 in G-ANC and 844 in I-ANC. Of the 1761, 260 in the G-ANC group were either lost to follow up or did not attend the G-ANC meetings, and 216 in the I-ANC group were lost to follow-up. There were no significant differences in age categories, maternal age, wealth index, number of previous pregnancies, and place of delivery between the two groups. 96% of the participants were either married/living together or cohabitating.The majority of participants had at least a secondary level of education, were Christians, and were not experiencing their first pregnancy, suggesting a relatively homogenous study population. The mean values for maternal age, wealth index, and number of previous pregnancies were similar across both groups.

Table 2 illustrates participants’ recognition of danger signs during pregnancy, comparing the two study arms (I-ANC and G-ANC) at two different time points (T0, or baseline, and T1, or 34 weeks gestation to 3 weeks post-delivery). At T1, women in the G-ANC arm were able to spontaneously list significantly more danger signs such as headache, vision changes, vaginal bleeding, leaking of fluids from vagina, convulsions, and swollen facecompared to women in the I-ANC arm. The cumulative 14-point Danger Signs Scale score averages increased for both groups at T1 compared to T0, however the increase was significantly higher for the G-ANC group compared to the I-ANC group (1.8 at T0 to 3.4 at T1 in the G-ANC arm compared to 1.7 at T0 to 2.2 at T1 I the I-ANC arm (*p* < 0.0001). Table 2 provides the count and percentages for the yes/no items and mean and standard deviation (SD) of the 14-point scale, and the *p*-values for the change over time for the two arms.

Table 3 illustrates preparations made for birth in both I-ANC and G-ANC groups at T0 and T1. At T1, the G-ANC group exhibited substantial improvements in preparedness compared to the I-ANC group: arranging for emergency transport, saving money, identifying someone to accompany the woman to the facility and identifying someone to care for other family members had the largest effects. The average cumulative Birth Preparedness 11-point scale score was significantly higher in the G-ANC group compared to the I-ANC group at T1 (1.4 at T0 to 2.9 at T1 in G-ANC arm vs. 1.4 at T0 to 1.7 at T1 in the I-ANC arm, *p* < 0.0001).

Table 4 illustrates respondents’ knowledge related to preventing problems before childbirth in the I-ANC and G-ANC groups at T0 and T1. At T1, the G-ANC group demonstrated significant improvements in recognition of preventive measures compared to the I-ANC group. Notably, there were substantial increases in women in the G-ANC group reporting recognition of the following preventive measures: sleeping under insecticide-treated bed nets andtaking malaria prophylaxis. The average of 11-point cumulative prevention scale scores was significantly higher in the G-ANC group at T1 compared to the I-ANC group (1.7 at T0 to 2.2 at T1 for I-ANC vs. 1.4 at T0 to 2.3 at T1 for G-ANC, *p* < 0.0001), indicating an increased recognition of preventive practices.

## Discussion

This study aimed to evaluate the impact of Group Antenatal Care (G-ANC) on Birth Preparedness and Complication Readiness (BPCR) among pregnant women in the Eastern Region of Ghana. BPCR included danger sign recognition and knowledge of preventive behaviors to improve birth outcomes. This study found that G-ANC significantly improved knowledge on the number of pregnancy danger signs that women could identify, as well as increasing the percentage of women who could identify individual danger signs such as headache.

G-ANC was also linked to an increase in the recognition of preventive measures, a cornerstone of maternal and child health. From the consistent use of insecticide-treated bed nets to adopting healthier dietary habits and avoiding alcohol, pregnant women in the G-ANC group recognized a range of practices aimed at ensuring their well-being and that of their unborn children. Prior studies of G-ANC demonstrated that integrating HIV prevention into health promotion, enhanced partner communication for healthy reproductive behaviors [15]. These findings, when viewed alongside the improved BPCR and danger sign recognition, paint a holistic picture of the positive impact of G-ANC on maternal healthcare. This is in keeping with several other studies that have demonstrated the value of G-ANC [16–19].

Improvements observed in both the control and intervention groups with regard to birth preparedness emphasize the importance of antenatal care in promoting safe childbirth practices, as both groups had increased knowledge and higher levels of birth preparedness following nine months of ANC. However, the intervention group, which received G-ANC, demonstrated significant advancements in several critical areas. There was a substantial increase in arranging emergency transport and saving money for transportation, ensuring timely access to healthcare facilities during childbirth. This is similar to findings from Lori et al. [14]. where G-ANC participants reported higher rates of discussing delivery locations with midwives, arranging emergency transport, and saving money for their birth [14]. Equally significant was the finding in this study that women in G-ANC were more likely to identify a companion for facility visits, indicating strengthened social support networks, a crucial component in maternal care. These findings align with research conducted in Kenya and Nigeria by Grenier et al. [19]. In a randomized controlled trial of G-ANC vs. I-ANC, the authors assessed the completion of individual BP/CR actions by a self-reported composite measure of seven BP/CR components: identified a facility, made a transportation plan, identified a companion, saved money, agreed on a decision-maker, agreed on an alternate decision maker, and prepared a birth kit. In both countries, women in the intervention arm were more likely than those in the control arm to complete all seven recommended BP/CR components [19].

The findings of this study provide compelling evidence of the effectiveness of G-ANC in improving women’s awareness of danger signs during pregnancy, including their ability to recognize potentially life-threatening symptoms. This heightened awareness, coupled with improved preparedness strategies, positions women in G-ANC to seek timely medical attention, ultimately reducing maternal and neonatal morbidity and mortality. This result compares to other studies conducted in central Tanzania where women who were aware of obstetric danger signs were more prepared for birth and complications, [20] and where conversely in Southern Ethiopia and India, women’s lack of knowledge led to poor pregnancy outcomes, in part due to limited emphasis on BPCR during routine ANC [21].

This study stops short of demonstrating significant differences in health outcomes associated with G-ANC. Other researchers have seen no difference in gestational duration, preterm birth, or cesarean section rates when comparing G-ANC and I-ANC [22], yet this may be partially attributable to the selection of healthy participants with low-risk pregnancies.

This study has several notable strengths. First, this was a rigorously designed, cluster-randomized controlled trial across 14 facilities representing urban and rural areas in Ghana, reflecting generalizability to other areas of Ghana. Further, the participants were identical in the balanced demographic characteristics distribution between the control and intervention groups, achieved through randomization, strengthens the external validity by minimizing potential biases, ensuring that the observed improvements are attributable to the intervention rather than external factors. This study also incorporated rigorous training of midwives, ongoing process evaluation and quality monitoring [23], and acute attention to the fidelity of the intervention [13].

Nonetheless, this study has several limitations. First, we asked women to list the danger signs and prevention strategies they could remember, but this may not equate to taking appropriate action. While knowledge and recognition are a necessary prerequisite to taking action in the face of a danger sign, knowledge may not be sufficient to ensure action. Future research that explores actions taken by the intervention group compared to the control group would strengthen the conclusions drawn about the impact of G-ANC. The second limitation is the challenge associated with asking women to self-report steps they have taken to prepare for birth, as social desirability may encourage them to exaggerate. Given the range of responses, we do not believe this is a serious limitation, but it is worth mentioning that self-reported data are not as reliable as behaviors that are directly observed. Finally, this study was designed for healthy pregnant women without known complications. Findings may be different if the study included women with pregnancy complications or high-risk pregnancies.

Despite its limitations, this study has several important implications. These findings emphasize the need for the widespread adoption of G-ANC as a standard of care for uncomplicated pregnancies. By empowering pregnant women with knowledge, enhancing their preparedness, and strengthening their support networks, G-ANC emerges as an important strategy to ensure safe pregnancies and improve maternal and child health outcomes. As the global community continues to strive for universal access to quality healthcare, the outcomes of this study can inform future interventions and policies toward the ultimate goal of ensuring the well-being of mothers and children worldwide Group antenatal care has transformative effects on birth preparedness, complication readiness, and preventive behaviors among pregnant women in the Eastern Region of Ghana and potential for similar results globally.

### Electronic supplementary material

Below is the link to the electronic supplementary material.

Supplementary Material 1

Supplementary Material 2

Supplementary Material 3

## Data Availability

Data are available through the University of Michigan’s permanent data repository, Deep Blue. Group Antenatal Care to Promote a Healthy Pregnancy and Optimize Maternal and NewbornOutcomes: A Cluster Randomized Controlled Trial (The GRAND project): https://doi.org/10.7302/d5ct-ne90 (Data are available at the bottom of the entry).
