# Supplementary material for: Effect of group antenatal care versus individualized antenatal care on birth preparedness and complication readiness: a cluster randomized controlled study among pregnant women in Eastern Region of Ghana
Source: BMC Pregnancy Childbirth. 2024 Aug 16;24:546. doi: 10.1186/s12884-024-06743-1 (PMC11328422; doi:10.1186/s12884-024-06743-1)
Supplement: Supplementary file 2 — Supplementary Material 2 [file 12884_2024_6743_MOESM2_ESM.pdf]

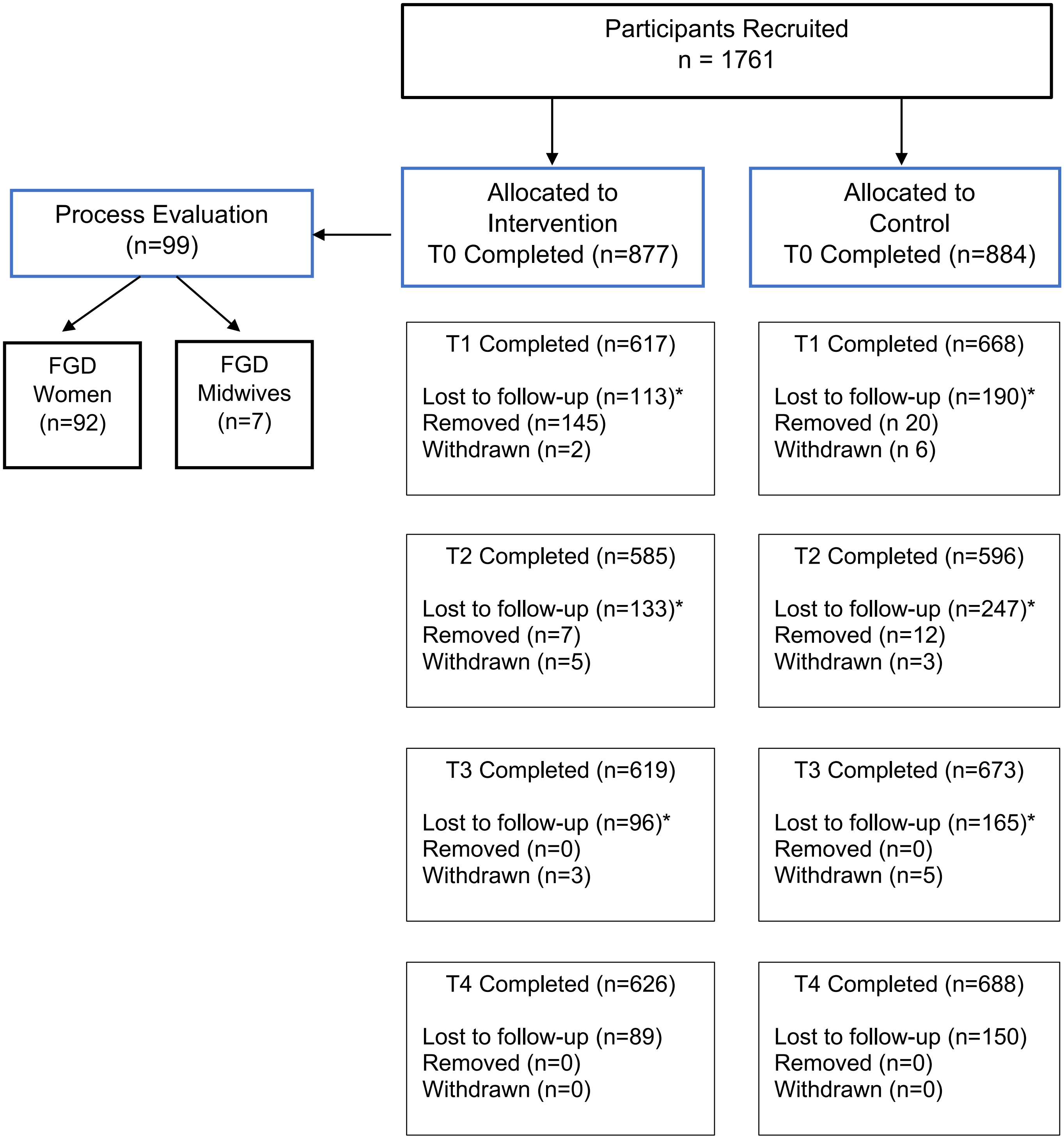

* Participants lost to follow-up at T1, T2, or T3 remain eligible to complete surveys at subsequent timepoints.
