# Supplementary material for: Effect of group antenatal care versus individualized antenatal care on birth preparedness and complication readiness: a cluster randomized controlled study among pregnant women in Eastern Region of Ghana
Source: BMC Pregnancy Childbirth. 2024 Aug 16;24:546. doi: 10.1186/s12884-024-06743-1 (PMC11328422; doi:10.1186/s12884-024-06743-1)
Supplement: Supplementary file 3 — Supplementary Material 3 [file 12884_2024_6743_MOESM3_ESM.pdf]

Protocol

Group Antenatal Care in Ghana: Protocol for a Cluster Randomized Controlled Trial

Jody R Lori[1*], MS, PhD; John E O Williams[2*], MBChB, MPH, MA, PhD; Vida A Kukula[2], BSc, MPH; Veronica E A Apetorgbor[2], BA, MPH; Elizabeth A Awini[2], PhD; Georgina Amankwah[2], BSc, MSc; Ruth Zielinski[1], PhD; Nancy Lockhart[1], BSN, MSN; Katherine H James[3], MHI; Cheryl A Moyer[3], MPH, PhD

[1]Department of Health Behavior and Biological Sciences, University of Michigan, Ann Arbor, MI, United States

[2]Dodowa Health Research Centre, Ghana Health Service, Dodowa, Ghana

[3]Department of Learning Health Sciences, University of Michigan Medical School, University of Michigan, Ann Arbor, MI, United States

[*]these authors contributed equally

Corresponding Author:
Jody R Lori, MS, PhD
Department of Health Behavior and Biological Sciences
University of Michigan
400 N. Ingalls Bldg, Suite 3320
Ann Arbor, MI, 48109
United States
Phone: 1 7347630097
Email: jrlori@umich.edu

Abstract

Background: While group antenatal care (ANC) has been delivered and studied in high-income countries for over a decade, it has only recently been introduced as an alternative to individual care in sub-Saharan Africa. Although the experimental design of the studies from high-resource countries have been scientifically rigorous, findings cannot be generalized to low-resource countries with low literacy rates and high rates of maternal and newborn morbidity and mortality. The Group Antenatal Care Delivery Project (GRAND) is a collaboration between the University of Michigan in the United States and the Dodowa Health Research Centre in Ghana. GRAND is a 5-year, cluster randomized controlled trial (RCT). Our intervention—group ANC—consists of grouping women by similar gestational ages of pregnancy into small groups at the first ANC visit. They then meet with the same group and the same midwife at the recommended intervals for care.

Objective: This study aims to improve health literacy, increase birth preparedness and complication readiness, and optimize maternal and newborn outcomes among women attending ANC at seven rural health facilities in the Eastern Region of Ghana.

Methods: Quantitative data will be collected at four time points using a secure web application for data collection and a database management tool. Data will be analyzed on an intention-to-treat basis to test the differences between the two arms: women randomized to group-based ANC and women randomized to routine individual ANC. We will conduct a process evaluation concurrently to identify and document patient, provider, and system barriers and facilitators to program implementation.

Results: The study was funded in September 2018. Recruitment and enrollment of participants and data collection started in July 2019. In November 2021, we completed participant enrollment in the study (n=1761), and we completed data collection at the third trimester in May 2022 (n=1284). Data collection at the additional three time points is ongoing: 6 weeks postpartum, 6 months postpartum, and 1 year postpartum.

Conclusions: This study is significant and timely because it is among the first RCTs to be conducted to examine the effects of group ANC among low-literacy and nonliterate participants. Our findings have the potential to impact how clinical care is delivered to low-literacy populations, both globally and domestically, to improve maternal and newborn outcomes.

Trial Registration: ClinicalTrials.gov NCT04033003; https://clinicaltrials.gov/ct2/show/NCT04033003

International Registered Report Identifier (IRRID): DERR1-10.2196/40828

(JMIR Res Protoc 2022;11(9):e40828) doi: 10.2196/40828

XSL•FO
RenderX

KEYWORDS

group care; antenatal care; group antenatal care; health literacy; maternal health literacy; Ghana; sub-Saharan Africa; care seeking; maternal outcomes; neonatal outcomes

Introduction

Background

In 2017, the maternal mortality ratio in Ghana was estimated to be 310 per 100,000 live births for the 7-year period prior to the Ghana Maternal Health Survey [1]. While 89% of women in Ghana surveyed had attended the minimum standard of four antenatal care (ANC) visits, 20% of women continued to give birth at home [1]. In contrast to the decline in infant and under-5 mortality, neonatal mortality has remained stagnant since 2007 [1].

ANC has the potential to play a pivotal role in ensuring positive pregnancy outcomes for both mothers and their newborns [2]. While ANC is widely available and attended by the majority of pregnant women in Ghana, the expected impact on birth outcomes is yet to be fully realized. Thus, it is vital to examine the way ANC is being delivered and to explore alternatives to the current model to enhance positive birth outcomes.

In addition to its clinical components, ANC is designed to teach pregnant women to recognize the danger signs that might warn them of complications that could affect either themselves or their babies, and to encourage prompt care seeking for such danger signs. ANC is also designed to promote a healthy lifestyle, to integrate positive health behaviors, and to develop a trusting relationship with a health care provider and the health system.

While group ANC has been delivered and studied in high-resource settings for over a decade, it has only recently been introduced as an alternative to individual care in sub-Saharan Africa. Two randomized controlled trials (RCTs) examining group ANC versus routine individual care conducted in the United States found that women assigned to group care had significantly better antenatal knowledge, had greater satisfaction with care, and were less likely to have a preterm birth than those in standard care. In addition, the trials showed more favorable birth, neonatal, and reproductive outcomes in the intervention groups [3,4]. Although the experimental design of the studies from high-resource countries are scientifically rigorous, findings cannot be generalized to low-resource countries with low literacy rates and high rates of maternal and newborn morbidity and mortality.

In sub-Saharan Africa, data from three pilot studies found ANC delivered in groups to be acceptable and feasible to both women and providers in Ghana, Senegal, Tanzania, and Malawi [5-7]. A two-country cluster RCT found a higher likelihood of birth in a health care facility for Nigerian women in group versus standard ANC and a higher frequency of ANC visits in both Kenya and Nigeria [8]. Finally, a large cluster RCT conducted in Rwanda to examine the impact of group ANC on gestational age at birth found no significant difference in gestational age between intervention and control groups.

This is a critical time during which to examine group ANC in order to promote healthy pregnancy and optimize maternal and newborn outcomes in low-resource settings [9]. This paper describes the design and evaluation plan for a cluster RCT that is powered to fill the knowledge gap in women's health literacy skills in order to increase self-care knowledge and care seeking during intrapartum and postpartum periods.

Description of the Model for Group Antenatal Care

The World Health Organization (WHO) Standards for Maternal and Neonatal Care [9] guided our iterative process with content experts from the United States, Ghanaian health care providers, pregnant women, and stakeholders to ensure local and cultural relevance. The group-based ANC model in this study was developed and tested for acceptability and feasibility by the corresponding author (JRL) and her Ghanaian team for the first time in a clinical setting in Ghana [5,10]. At the core of the model is a negotiation process acknowledging that some health messaging may be in conflict with cultural beliefs. The model allows participants to incorporate safe, feasible, and culturally acceptable health beliefs into self-care actions by being inclusive of traditional practices that are not harmful. As part of the model, participants and the facilitator "agree" on safe and acceptable actions within the context of the setting that are then practiced by the group.

At the initial ANC visit, women are placed into small groups of 10 to 14 women with pregnancies of similar gestational age. Standard complete histories and physical exams as well as lab tests are completed, with group visits starting at the second ANC visit. Prior to the start of each group, blood pressure and weight are measured and a urinalysis is performed by each woman with help from the midwife. Each woman then receives an individual assessment with the midwife to measure fundal height, listen to fetal heart tones, and answer any questions she prefers not to raise in the group. The midwife and women then sit in a circle facing one another for a 60- to 90-minute facilitated discussion. The model uses strategies such as storytelling, peer support, demonstration, and teach-back to enhance its effectiveness. Health literacy is incorporated as an integral part of clinical practice within the model, not as an add-on to care.

Evidence-based information is presented in a nonhierarchical, patient-centered, participatory way. Picture cards (Figure 1) are used to enhance communication and learning in the group setting. They provide a mechanism to help convey new concepts and ideas.

The picture cards encourage valuable group discussion and are an educational aid to stimulate thinking and reflection, dialogue, and learning among participants. Content is repeated multiple times in a variety of ways to enhance retention, including the following: (1) auditory (ie, listening to stories and signs of problems), (2) visual (ie, through the use of demonstration and picture cards), and (3) kinesthetic (ie, practicing actions and handling picture cards).

XSL•FO
RenderX

The Facilitator's Guide for Group Antenatal Care, developed by JRL, provides a step-by-step guide that details how to conduct each of the group ANC visits, become a facilitator, enhance adult learning, provide respectful maternity care, and monitor for program quality, performance, and fidelity.

Figure 1. Examples of picture cards.

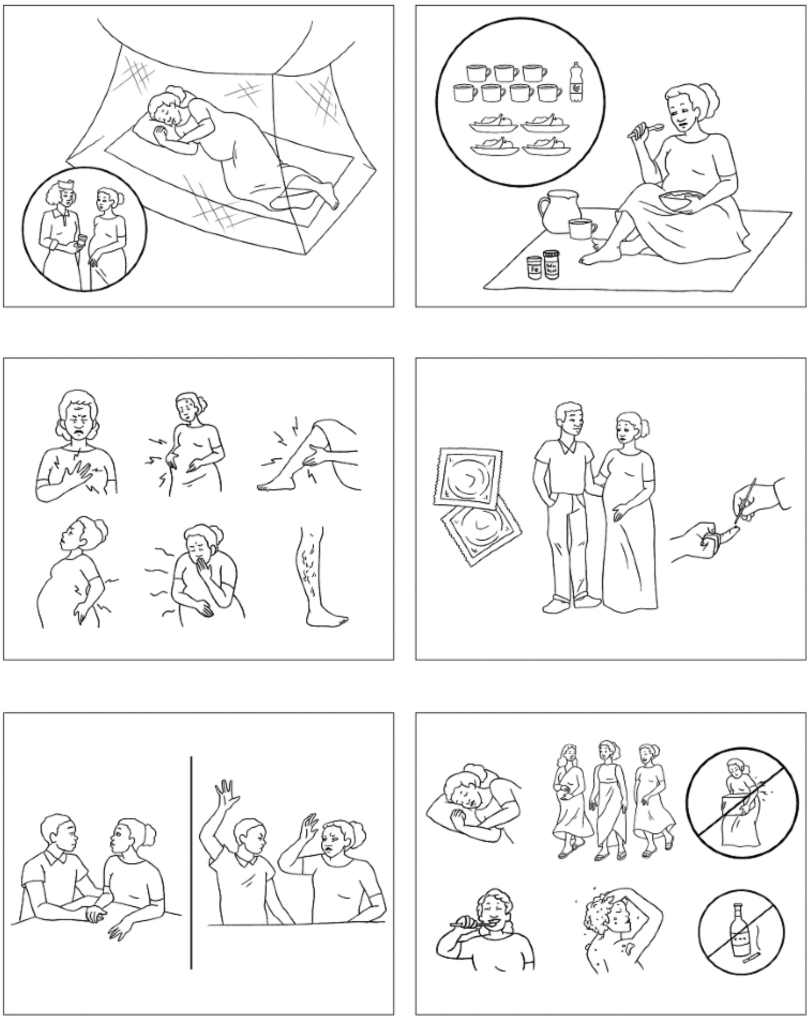

Aims and Objectives

The Group Antenatal Care Delivery Project (GRAND) is designed to improve health literacy, increase birth preparedness and complication readiness, and optimize maternal and newborn outcomes among women attending group-based ANC at seven rural health facilities serving predominantly low-literacy and nonliterate pregnant women in the Eastern Region of Ghana. More specifically, GRAND is designed to achieve the following aims:

- Aim 1: to quantify differences in birth preparedness and complication readiness, including knowledge of danger signs and recommended action steps, between women randomized to group-based ANC and those randomized to routine individual ANC.
- Aim 2: to assess behavioral differences in care-seeking patterns (eg, facility birth rates, postnatal care, and postpartum care) between women randomized to group-based ANC and those randomized to routine individual ANC.
- Aim 3: to evaluate the clinical outcomes of mothers and their newborns (eg, decrease in maternal morbidities and perinatal and neonatal mortality) between women randomized to group-based ANC and those randomized to routine individual ANC immediately postpartum and up to 1 year following birth.

We hypothesize that pregnant women randomized into group-based ANC, as compared to women who received routine individual ANC, will exhibit increased health literacy through the following: (1) increased birth preparedness, including recognition of danger signs and knowledge of how to respond to such signs; (2) higher rates of care-seeking behaviors, including seeking care for problems identified during pregnancy, higher facility delivery rates, and increased attendance at postnatal and postpartum care; and (3) better clinical outcomes for themselves and their newborns.

XSL•FO
RenderX

Methods

Overview

This study uses a theoretical model originally developed by Squiers et al [11] and modified in our preliminary research to assess maternal health literacy [5]. The Health Literacy Skills Framework uses an ecological perspective to help assist in the development and testing of potential interventions to impact a patient's health literacy [11]. As illustrated in Figure 2, our modified theoretical model, which is renamed the Maternal Health Literacy Skills Framework [5], is used to guide the aims and data analytic plan.

Our model addresses how group ANC builds knowledge by increasing the comprehension of stimuli, promoting self-determination, increasing action, and ultimately improving maternal health behaviors and outcomes. It considers how the individual's comprehension of stimuli and potential mediators may impact overall health behaviors and outcomes.

Figure 2. Modified Maternal Health Literacy Skills Framework.

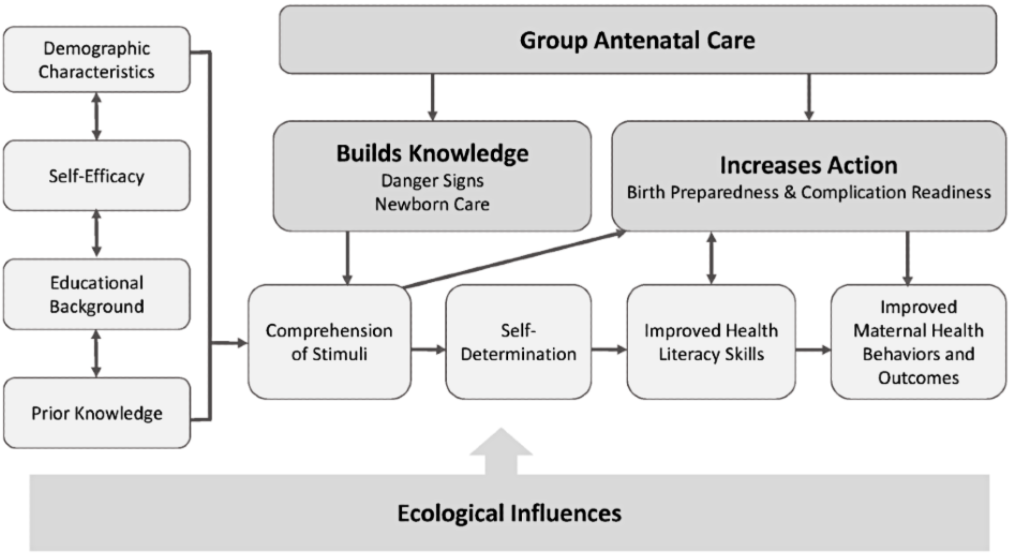

Study Design

GRAND is a 5-year cluster RCT. The study was registered at ClinicalTrials.gov (NCT04033003) on July 25, 2019, and is a collaboration between the University of Michigan in the United States and the Dodowa Health Research Centre in Ghana. Health facilities were selected based the number of ANC registrants per month and the average gestational age of pregnancy among women at registration in each facility. Facilities were then matched based on facility type, district, and number of monthly ANC registrants.

Study Setting

The study setting for GRAND includes four districts—Akwapim North, Yilo Krobo, Nsawam-Adoagyiri, and Lower Manya Krobo—within the Eastern Region of Ghana. Ghana (Figure 3) has a population of approximately 30 million people and is situated in Western Africa between Togo, Burkina Faso, Ivory Coast, and the Atlantic Ocean.

Ghana is divided into 16 administrative regions, with the Eastern Region situated north and adjacent to the region that includes the capital city of Accra, the Greater Accra Region. While Greater Accra is predominantly urban and periurban, the Eastern Region relies on a primarily agrarian economy, including both subsistence and commercial farming. Approximately 20% of residents never attended any formal schooling, with another 60% stopping their education at the primary level (14.5%) or at the junior secondary (ie, high school) level (45.3%). Women are twice as likely as men to have never received any schooling [12]. According to the 2017 Ghana Maternal Health Survey, the fertility rate for the region is 3.8, comparable to the national average of 3.9 [1].

XSL•FO
RenderX

Figure 3. Map of study districts in Ghana.

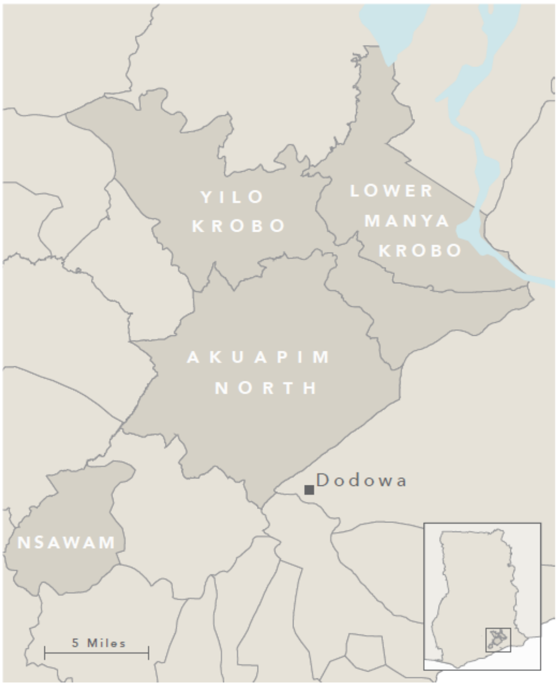

Sampling and Randomization Frame

Facilities were randomized using a matched-pair method. Variables for matching included the number of deliveries and the average gestational age of pregnancy among women at the time of enrollment for ANC in each facility, so that facilities within each pair are similar to each other with regard to these matching factors. For each pair of facilities, one site was randomly assigned to group-based ANC (intervention) and the other to routine individual ANC (control). The matching and randomization process was completed using the nbpMatching package from R software (version 1.5.0; R Foundation for Statistical Computing) [13]. The locations of the chosen facilities ensures that participating facilities will be far enough apart to minimize the likelihood of cross-group contamination.

Power and Sample Size Calculations

We calculated the sample size based on three primary outcomes: the change in birth preparedness, complication readiness index scores, and the percent change in women obtaining maternal postpartum checkups and babies obtaining postnatal checkups within first 2 days after birth. See Table 1 for a complete list of primary and secondary outcomes.

According to the literature, the median intraclass correlation coefficient (ICC) was 0.010 [14]. Since we proposed a cluster randomized design based on seven intervention facilities and seven control facilities, we considered the effect of the ICC. The ICC is a measure of the extent to which the effect of the intervention differs across facilities. Hence, we conducted our sample size calculation for an ICC equal to 0.01 using the CRTSize package from R software [15]. First, the percentage of women in Ghana who were categorized as "prepared for birth" was 30% [16]. We expect that our ANC intervention will improve the preparedness to 45%, as measured by the birth preparedness and complication readiness index. At a significance level of .05, we need 84 women per facility to reach 80% power to detect such an effect. Next, approximately 60% of women in the Eastern Region of Ghana receive a maternal postpartum checkup in the first 2 days after birth [12]. We expect that our intervention will increase this value to 75%. To test this, we need 76 women per facility. Finally, the current percentage of newborns obtaining postnatal checkups in rural Ghana within 2 days is 22% [12]. We expect that our intervention will increase this value to 35%, in which case we will need at least 100 women per facility to see such an effect. To preserve power due to attrition, we proposed recruiting 120 women per facility. Hence, the total number of women to be recruited is 1680 based on an attrition rate of 20% in our pilot work.

XSL•FO
RenderX

Table 1. Primary and secondary outcomes.

Aims	Primary outcomes	Secondary outcomes
Aim 1: to quantify differences in birth preparedness, knowledge of pregnancy and newborn danger signs, and recommended action steps	• Ability to identify danger signs in pregnancy (eg, bleeding, severe headache, blurred vision, and fever)<br>• Birth preparedness and complication readiness (eg, saved money, identified birth facility and emergency transportation to facility, and identified blood donor)<br>• Ability to identify newborn danger signs (eg, poor suck, jaundice, difficulty or fast breathing, and convulsions)	• Ability to identify postpartum danger signs (eg, increased bleeding or large clots, weakness or fainting, fever, pain in abdomen or breasts, and painful urination)<br>• Ability to identify the recommended action steps when a problem is identified (eg, call for help, have a plan for transportation, identify someone to accompany you to the facility, identify someone to care for the family, go straight to the facility, and supportive care along the way to the facility)<br>• Self-efficacy, operationalized care-seeking history, and health information knowledge
Aim 2: to assess behavioral differences in care-seeking patterns (eg, facility birth rates, postnatal care, and postpartum care)	• Attendance at 4 or more ANC[a] visits<br>• Facility birth<br>• Four postnatal or postpartum checkups for both mother and newborn in the first 6 weeks after birth	• Uptake of modern family planning methods at 6 months postpartum<br>• Infant immunized per EPI[b] scheduled at 1 year<br>• Completion of IPTp2[c] malaria prophylaxis during pregnancy
Aim 3: to evaluate the clinical outcomes of mothers and their newborns (eg, decrease in maternal morbidities and perinatal and neonatal mortality)	• Maternal pregnancy-related morbidities (eg, puerperal sepsis and delayed postpartum hemorrhage)<br>• Birth outcome (eg, stillbirth, live birth, and early neonatal mortality)	• At least 2 tetanus toxoid vaccines during ANC<br>• Infant protected against neonatal tetanus<br>• Hemoglobin level upon hospital admission, dichotomized as normal or anemic (<9.5 g/dL)<br>• Infant birth weight (normal vs low [<2500 g])<br>• Repeat pregnancy within 1 year<br>• Exclusive breastfeeding at 6 months

[a]ANC: antenatal care.

[b]EPI: Expanded Program on Immunization.

[c]IPTp2: intermittent preventive treatment of malaria for pregnant women.

Trainings

Training of Research Personnel

Prior to data collection, all research assistants (RAs) were trained for the study by the primary investigator and coinvestigators. All trainings were held in English, the official language of Ghana, with discussions regarding key terms in Dangbe, Ga, Akan, and Ewe. Trainings included the following: (1) an overview of the study and its protocol, (2) information about the ethical treatment of human subjects, (3) standardized record keeping and data collection for the study, and (4) strategies for reducing bias and error. Biannual refreshers will be conducted with all RAs. All RAs are fluent in English as well as in the dialect and culture of their assigned area.

Training of Clinical Personnel

Prior to data collection, we conducted a training of trainers for research personnel at the Dodowa Health Research Centre and maternal, newborn, and child health nurses representing the four District Health Directorates. All registered nurses and registered midwives providing ANC at both intervention and control facilities received an update on the essential components of ANC based on WHO guidelines to ensure equal quality at all sites at baseline. Providers at intervention sites were trained to implement group-based ANC, whereas providers at control sites will continue delivering routine individual ANC. Providers at study sites randomized to group care were trained in the delivery of the methodology. The provider training mirrors the facilitator's guide, including an emphasis on active listening, ideal conditions to maximize learning, and the use of picture cards as an important training resource for low-literacy learners. These trainers, with assistance from the primary investigator and two experienced trainers, then conducted a 3-day didactic training with groups of 10 to 12 clinical personnel focused on facilitating group ANC, use of the methodology, organizing groups, and an overview of the research. All trainings were in English, the official language of Ghana, with discussions regarding key terms in both English and the local languages. Participants then practiced delivering care using the group model with support from the trainers. A learning methods checklist and a fidelity checklist for provider readiness, which was established during preliminary studies, were used to provide feedback to participants during practice and to establish when each individual is ready to take on facilitating a group, based on the checklist scores.

Recruitment of Participants and Informed Consent

Recruitment of women will occur at individual health facilities. The trained RA works with clinic staff to identify women who meet the eligibility criteria and are healthy enough to discuss enrolling in an ANC intervention. The RA will inform health facility staff as to when they will be at the clinic and available to women interested in learning more about the study. Midwives will identify women (1) whose pregnancies are at less than 20 weeks' gestation; (2) who speak Dangme, Ga, Akan, Ewe, or English; (3) who are over the age of 15 years; and (4) who are not considered high risk.

XSL•FO
RenderX

The midwife will then instruct women who qualify to talk to the RA if they are interested in learning more about the study. Women who approach the RA will be read an approved recruitment script. Those who are willing to participate will be taken through an informed consent procedure and complete baseline data collection.

The procedure for informed consent includes the following:

1. An informed consent document in English is translated into Dangme, Ga, Akan, and Ewe.
2. The informed consent document is read aloud individually to all potential participants in private.
3. The Ghanaian RA asks the potential participant questions to ensure understanding of the research process and informed consent document and invites questions until the information is clear.
4. The participant signs the document or marks it with a thumbprint.
5. The RA uses the camera on the encrypted tablet to take a photograph of the signed or thumbprinted page; the image is then stored securely, similar to all study data.

In the Eastern Region, 10.4% of women and girls aged 15 to 49 years have never attended school, and only 15.7% have completed secondary school or higher [12]. A teach-back method will be used to confirm participant comprehension of the study requirements and methodology. The RA will ask potential participants to describe their understanding of the study's purpose, procedure, risks, and benefits using open-ended prompts and will repeat the material until understanding is achieved.

Data Collection and Measures

All quantitative data will be collected by trained RAs using encrypted and password-protected tablets as well as a secure web application for data collection and database management geared to support online and offline data capture for research studies. When an internet connection is not available, data will be collected offline and stored on the encrypted tablet. Once a connection is available, these data will be uploaded, verified for accuracy and completeness, and stored on a secure server.

No data will be collected by clinical providers. Data collection will occur at five time points in both intervention and control arms (see Table 2 for measurements at each time point):

1. Time point 0: the baseline session occurs immediately following the consent processes. Data are collected by trained RAs using a structured survey; health information is self-reported.
2. Time point 1: this session occurs at 34 weeks' gestation to 3 weeks postdelivery. Data are collected by trained RAs using a structured interview and by retrieving data from the ANC card.
3. Time point 2: this session occurs 6 to 12 weeks after delivery. Data are collected by trained RAs using a structured interview and by retrieving information from ANC cards on maternal and newborn clinical outcomes using a predetermined set of indicators.
4. Time point 3: this session occurs 5 to 8 months postpartum; data are collected via phone by trained RAs using a structured interview.
5. Time point 4: this session occurs 11 to 14 months postpartum; data are collected via phone by trained RAs using a structured interview.

During visits, midwives record clinical health–related outcomes (ie, place of birth, hemoglobin levels, newborn birth weight, maternal and newborn morbidities, stillbirth, and postpartum visit within 2 days postbirth) on the women's ANC cards. These data will be collected by the RA postdelivery.

XSL•FO
RenderX

Table 2. Measures and time points.

Time point and domains	Measure or source		
	Aim 1	Aim 2	Aim 3
**Time point 0: enrollment**			
1. Demographic characteristics	Baseline survey; section I: demographics	Baseline survey; section I: demographics	—[a]
**2. Self-efficacy**			
Extracting health information	Baseline survey; section II: self-efficacy	Baseline survey; section II: self-efficacy	—
Care-seeking history	Baseline survey; section II: self-efficacy and contraceptive self-efficacy scale	Baseline survey; section II: self-efficacy and contraceptive self-efficacy scale	—
**3. Educational background**			
Level of education	Baseline survey; section I: demographics	Baseline survey; section I: demographics	—
**4. Prior knowledge**			
Ability to identify danger signs, birth preparedness, and complication readiness	Baseline survey; section III: birth preparedness and complication readiness	Baseline survey; section III: birth preparedness and complication readiness	—
5. Health literacy skills	Maternal health literacy index	Maternal health literacy index	—
6. Ecological influences	Baseline survey; section I: demographics	Baseline survey; section I: demographics	—
**Time point 1: third trimester**			
7. Comprehension of stimuli	Third-trimester questionnaire	Third-trimester questionnaire	—
**8. Self-determination**			
Intent to use family planning and preparation for birth	Third-trimester questionnaire	Third-trimester questionnaire	—
Two or more tetanus toxoid vaccines and completion of IPTp2[b] malaria prophylaxis	ANC[c] card	ANC card	—
**Time point 2: postbirth**			
9. Clinical health–related outcomes	—	—	ANC card
**10. Self-determination**			
Attendance at 4 or more ANC visits	ANC card	ANC card	—
Adherence to care	Number of ANC visits	Number of ANC visits	—
11. Health-related behavior	—	—	Questionnaire for women who recently delivered, up to 6 weeks postpartum
12. Clinical health–related outcomes	—	—	Questionnaire for women who recently delivered, up to 6 weeks postpartum
**Time point 3: 6 months postpartum**			
13. Health-related behavior	Maternal health literacy index	Maternal health literacy index	6-month postpartum survey
14. Clinical health–related outcomes	—	—	6-month postpartum survey
**Time point 4: 1 year postpartum**			
15. Health-related behavior	—	—	1-year telephone survey
16. Clinical health–related outcomes	—	—	1-year telephone survey

[a]The data collected in the domain do not contribute to the research objectives of the aim.

XSL•FO
RenderX

[b]IPTp2: intermittent preventive treatment of malaria for pregnant women.
[c]ANC: antenatal care.

Process Evaluation

Overview

We will concurrently conduct a process evaluation to identify and document patient, provider, and system barriers and facilitators to program implementation. Using both quantitative and qualitative methods, we will identify potential and actual influences on the quality and conduct of the program's operations, implementation, and service delivery. We will employ structured observations of group sessions, interviews with providers, focus groups with women, and tracking logs to record how the intervention is delivered and received, document program fidelity, and identify opportunities to enhance the delivery of the intervention, while maximizing consistency in intervention delivery across sites. This process evaluation will add value to the analysis of the group ANC intervention by identifying barriers and facilitators at multiple levels throughout the study. For this process evaluation, we will focus on both fidelity of the intervention and dose, or frequency.

Individual Interviews With Midwives

All midwives involved in the intervention arm will be asked to participate in the process evaluation. Midwives will be approached by a member of the research team at the end of the seventh group meeting and asked if they are interested in providing feedback about group care. Those willing to participate will be taken through a consent process before the first interview begins. Each midwife will be interviewed at two random times, and each interview will last approximately 40 minutes. A structured interview using open-ended questions will be conducted to explore the midwife's perceptions of group versus standard ANC, barriers to implementation, challenges to integrating group-based ANC into the existing clinic workflow, and strategies that have helped with implementation. Interviews will be audiotaped, with permission from the participant, to ensure accuracy of responses; midwives can refuse to be audiotaped yet continue with the interview. The RA will write short-answer responses on a data collection form. Audiotapes will be transcribed and deidentified; tapes will be destroyed immediately after transcription. We have seven health facilities randomized to the intervention arm and 2 to 4 midwives at each facility; as each midwife may be interviewed twice, we expect that approximately 56 midwives may participate in the process evaluation.

Focus Group Discussion With Participants

Groups of women in the intervention arm will be randomly selected to participate in focus groups for process evaluation. We anticipate 10 random groups of 10 women through the course of the study for a total of 100 women in the focus groups. Women will be asked to describe their perceptions of group versus standard ANC, their perceptions of the value of group ANC, and how they could envision the process being improved.

Focus group discussions will be led by a member of the research team with randomly selected groups of women completing group ANC throughout the study. Focus groups participants will provide consent individually before they enter the focus group room so they may choose whether they want to participate. The group will be conducted in a private setting, and names will not be used during the discussion. Audiotapes will be transcribed and deidentified; tapes will be destroyed immediately after transcription. Each focus group discussion will last about 1 hour.

Structured Observations

A sample of 2 out of 7 group ANC visits will be observed for each provider to monitor fidelity to the model (eg, whether content is delivered as intended, women are engaged enough to actively participate in group discussions and activities, picture cards are used as written in the facilitator's guide, and feedback is provided to participants during demonstrations).

Tracking Logs

A brief form will be completed by the midwife provider each time an ANC visit is held to track the date of the session and the number of participants from the group in attendance, in order to track dose. See Figure 4 for flow diagram of enrollment and data collection.

XSL•FO
RenderX

Figure 4. Flow diagram of enrollment and data collection. ANC: antenatal care; FGD: focus group discussion; T0: time point 0 (baseline); T1: time point 1 (34 weeks' gestation to 3 weeks postdelivery); T2: time point 2 (6-12 weeks after delivery); T3: time point 3 (5-8 months postpartum); T4: time point 4 (11-14 months postpartum).

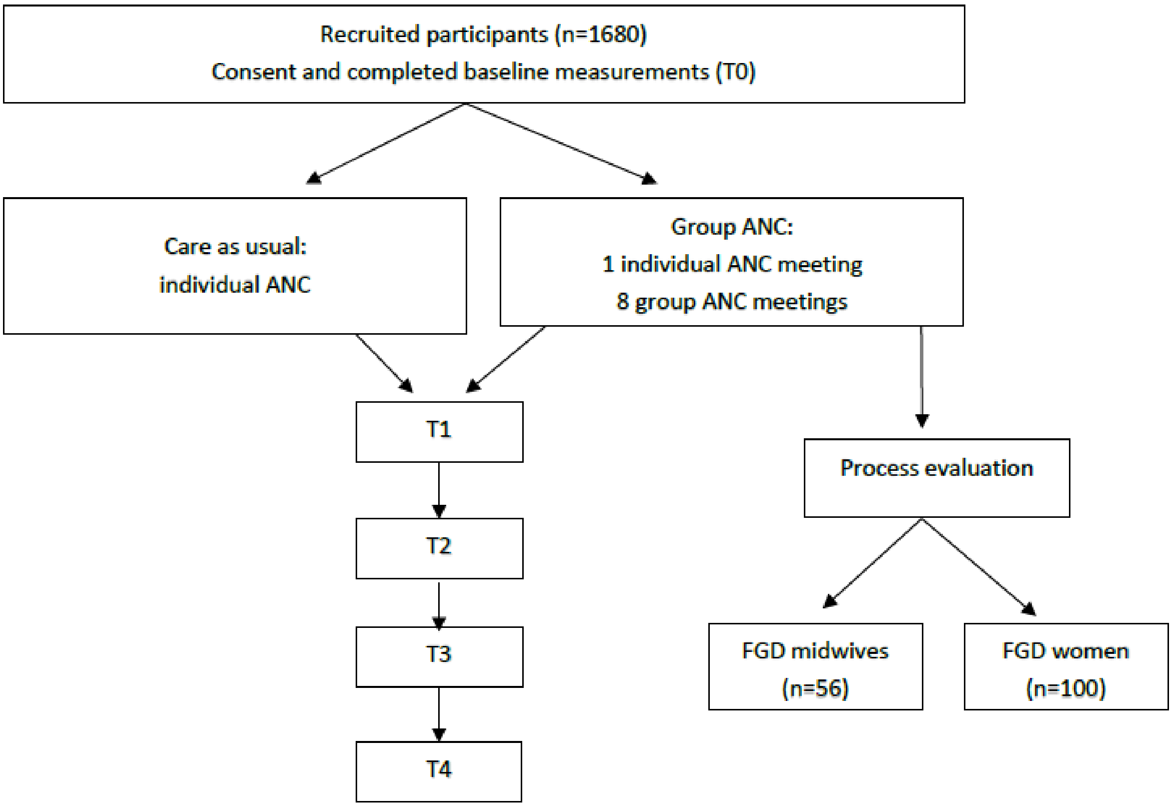

Data Analysis: Aims 1 to 3

Data from all participants randomly assigned to the intervention or control groups will be analyzed on an intention-to-treat basis. Deviations from randomized allocation will be reported. We will also conduct per-protocol analysis by eliminating noncompliers in the analysis. Summary statistics based on mean, SD, or frequency will be used to characterize the sample distribution of each arm. Proper transformations will be investigated and taken if the sample distributions of continuous variables violate the normality assumption. For aims 1 to 3, generalized linear mixed models will be used to test the differences between the two arms since a cluster RCT design will be used. There are four components in generalized linear mixed models: outcome variable, fixed effects, random effect, and link function. The fixed effects include an explanatory variable and covariates. In this study, all three aims have the same explanatory variable, which is a binary variable indicating the arm to which women are assigned. The study sites, gestational age, and women's demographic variables, such as education or literacy, marital status, pregnancy history, and medical history, will be added to the generalized linear mixed models as covariates to increase the precision of the estimates. The random effect is comprised of the 14 facilities. In this study, a random intercept model will be used to account for the cluster effect. The outcome variables and link functions in generalized linear mixed models depend on the aims and are described below. The construct of the generalized linear mixed models is to test whether the explanatory variable is significant at the level of .05 using the likelihood ratio test.

For aim 1, to quantify differences in the recognition of pregnancy and newborn danger signs and knowledge of recommended action steps, the birth preparedness and complication readiness index will be measured at enrollment and at the third trimester. We will add baseline data as covariates. The logit link function will be used for each binary birth preparedness and complication readiness question to test the efficacy of the group-based ANC method. The identity link function will be used when the outcome variable is a summary statistic of the birth preparedness and complication readiness index. When the *P* value of the explanatory variable is less than .05, we will declare a significant difference between the two arms. Average changes in birth preparedness and complication readiness summary statistics or odds ratios for each question will be used to quantify the effect of the group-based ANC intervention.

For aim 2, where we will assess behavioral differences in care-seeking patterns between the two arms, the outcome variables are frequency of attendance of ANC visits, facility birth, and postnatal or postpartum care. For the attendance outcome variable, the identity function will be used. For facility birth and postnatal or postpartum care, the logit link function will be used. When the *P* value of the explanatory variable is less than .05, we will declare a significant difference between the two arms. The effect of group-based ANC on attendance will be quantified by the average difference. The effects of group-based ANC on facility birth and postnatal or postpartum care will be quantified by odds ratios. For the secondary outcomes in aim 2, the logit or identity link function will be used in a way similar to the primary outcomes.

XSL•FO
RenderX

For aim 3, in order to evaluate the clinical outcomes of mothers and their newborns, the outcome variables are maternal pregnancy-related morbidities and newborn birth status. For maternal morbidities, the logit link function will be used. Since newborn birth status is classified into three categories—stillbirth, live birth, and early neonatal mortality—the cumulative logit link function [17] will be used. When the explanatory variable is significant at .05, the effect of group-based ANC on maternal pregnancy-related morbidities and newborn birth status will be interpreted using the odds ratio. We will illustrate the difference between outcomes using odds ratios for each pair of newborn birth statuses. The secondary outcomes will be analyzed similarly using identity or logit link functions.

For multiple outcomes in the same family, we will conduct direct inference using the Holm multiple testing procedure [18] to control for the family-wise error rate at a level of .05 [19]. The generalized linear mixed model analysis will be carried out using the lme4 package from R software [20]. All findings will be reported using the CONSORT (Consolidated Standards of Reporting Trials) statement as a guide [21]. Full transparency will be provided when reporting experimental details so that others may reproduce and extend our findings.

Data Analysis: Process Evaluation

The approach by Steckler et al [22] will guide the analysis of our process evaluation of the data. Qualitative data will be obtained from semistructured interviews. All qualitative data will be collected by the research team and will be transcribed verbatim into English, leaving key phrases that are difficult to translate intact, with the closest approximate meaning put into parentheses in the transcript. No data will be collected by clinical providers. All transcripts will be stored on a password-protected server. All data from semistructured interviews will be entered into NVivo qualitative software (QSR International) to assist with the identification of key themes. Structured observations will be recorded and summarized for key points. The use of an audit trail composed of methodological and analytical documentation and validation with colleagues will be used to achieve validity.

Ethics Approval

This study and all procedures were approved by the Institutional Review Boards (IRBs) at the University of Michigan (HUM-00161464) and the Ghana Health Service (GHS-ERC016/04/19). This is a report of a study protocol; therefore, human subject consent was not necessary. As required by the University of Michigan, regardless of the country of residence, all research staff, including principal investigators, coinvestigators, and RAs, on research projects that involve human study participants must complete the online program for education and evaluation in responsible research and scholarship or equivalent, and they must have their human subjects certification renewed every 3 years.

Results

The study was funded in September 2018. During the first year, we completed the following:

1. Developed a detailed research protocol.
2. Submitted the research protocol for full board approval to the University of Michigan, the Dodowa Health Research Centre, and the Ghana Health Service.
3. Received IRB approval with contingencies.
4. Updated the facilitator's guide and training materials for providers to reflect the new WHO Recommendations on Antenatal Care for a Positive Pregnancy Experience [9].
5. Hired and trained 6 Ghanaian RAs in the ethical conduct of research, the data collection protocol, and the use of research electronic data capture (REDCap) [23,24] for secure data management.
6. Developed and pilot-tested data collection instruments with modifications for the local context.
7. Identified study sites for inclusion.
8. Randomized study sites.

Study data are collected and managed using REDCap at the Dodowa Health Research Centre. REDCap is a secure, web-based software platform designed to support data capture for research studies. REDCap provides (1) an intuitive interface for validated data capture, (2) audit trails for tracking data manipulation and export procedures, (3) automated export procedures for seamless data downloads to common statistical packages, and (4) procedures for data integration and interoperability with external sources [23,24].

We also conducted a 3-day training for 10 champion trainers: 2 from each district in the research study and 2 from the provincial headquarters. The training covered an introduction to the study, an update or refresher on Ghanaian guidelines for ANC, and how to conduct group ANC using the facilitator's guide and methodology. A learning methods checklist was employed to ensure fidelity to the model. A schedule was prepared for the next 2 weeks of training at the district levels.

Recruitment and enrollment of participants and data collection started in July 2019. In November 2021, we completed participant enrollment in the study (n=1761), and we completed data collection at the third trimester in May 2022 (n=1284). Data collection at the additional three time points is ongoing: 6 weeks postpartum, 6 months postpartum, and 1 year postpartum. We are currently conducting preliminary data analysis and expect the results to be published in 2023.

Discussion

Overview

We hypothesize that pregnant women randomized into group-based ANC will exhibit increased birth preparedness and complication readiness, including recognition of danger signs and knowledge of how to respond to such signs. This may result in higher rates of care-seeking behaviors, including seeking care for problems identified during pregnancy, higher facility-based delivery rates, and increased attendance at postnatal and postpartum care appointments.

This study is significant and timely because it is the first cluster RCT to be conducted in Ghana to examine the effects of group-based ANC on maternal and newborn clinical and behavioral outcomes. Ghana is one of 24 priority countries targeted by the United States Agency for International

XSL•FO
RenderX

Development to improve maternal and child health and end preventable death [25].

Recent recommendations by the WHO call for rigorous research into group ANC to improve the use and quality of care [9]. A strength of our study is the use of a theoretical framework to examine health literacy. Initially considered as a patient's ability to read and understand written information, health literacy is now more broadly defined as a person's ability to acquire or access information, understand it, and use the information in ways that promote and maintain good health [26,27]. Despite a burgeoning emphasis on health literacy in high-resource countries [28], there are a dearth of studies examining interventions to improve health literacy in low-resource settings [29]. Even fewer studies have examined maternal health literacy, defined as the "cognitive and social skills which determine the motivation and ability of women to gain access to, understand, and use information in ways that promote and maintain their health and that of their children" [29]. New approaches to improve health literacy are sorely needed in countries where women and newborns continue to die from preventable causes [30].

Our process evaluation will allow us to contribute to a growing body of evidence that identifies barriers and facilitators to the implementation of group ANC. Findings from the process evaluation will contribute to eventual scale-up of the intervention in Ghana should group ANC be shown to improve maternal and newborn outcomes.

Our research team is committed to disseminating the findings from this proposed study in four different ways: (1) presentations at national and international conferences; (2) journal articles in peer-reviewed journals, including open access for our international colleagues; (3) community presentations, media events, and other public venues where we intend to discuss our findings; and (4) meetings and presentations with the Ghana Health Service to discuss cost-effective ways for scaling up the project and ensuring sustainability.

Limitations

Although we have designed a rigorous cluster RCT, neither the study sites nor the participants are blinded to the study conditions because providers at sites have been trained to deliver group ANC. We eliminated selection bias by randomly selecting sites using a stratified random sampling method from the sampling package in R software. Participating sites are limited to one rural area of Ghana; thus, results may not be generalizable to urban settings. However, results could guide country-wide policies for improving maternal and newborn health, and results could highlight the benefits of group ANC for similar rural areas across Africa where maternal and newborn morbidity and mortality are high.

Improving maternal and newborn health outcomes has been a major focus for the governments of many low- and middle-income countries, including Ghana. Free maternal and child health has been introduced in Ghana as part of a comprehensive policy to improve maternal health care delivery and reduce maternal and child deaths [1]. Group ANC has the potential to improve the quality of care and pregnancy outcomes for women and their newborns. Findings from this study will provide strong evidence and lessons learned to contribute to future policies and scale-up for all of Ghana.

Acknowledgments

We thank Mary Carpenter, Josephine Nowak, Mackenzie Dwyer, and Emily Johnson for their contributions to the development of the group antenatal care facilitator's guide and training manual. This study is funded by the National Institutes of Health (NIH), Eunice Kennedy Shriver National Institute of Child Health and Human Development (grant RO1HD096277). The development of the Facilitator's Guide for Group Antenatal Care was supported by a research grant from Fogarty International, NIH (grant 1 K01 TW008763-01A1; principal investigator JRL). The content is solely the responsibility of the author and does not necessarily represent the official views of Fogarty International or the NIH.

Data Availability

We will comply with all National Institutes of Health (NIH) policies on data sharing. Full transparency in reporting experimental details will be given so that others may reproduce and extend our findings. Deidentified data will be shared in Deep Blue for those wishing to conduct a secondary analysis of the data. Deep Blue is the University of Michigan's permanent, safe, and accessible service for providing access to the work conducted by researchers at the University of Michigan. The repository has data access policies and procedures consistent with NIH data sharing policies. Submitted data will conform to relevant data standards. Data will be deposited within 1 year of completion of the funded project period for the award or upon acceptance of the results for publication. We will identify where the data will be available and how to access the data in any publications and presentations authored or coauthored regarding these data, as well as acknowledge the repository and funding source in any publications and presentations.

Authors' Contributions

JRL, JEOW, and CAM were involved in all aspects of conceptualization and study design. JRL, CAM, and NL wrote the initial draft of the Group Antenatal Care Deliver Project protocol that was submitted for ethical review, with input from JEOW, VAK, VEAA, EAA, RZ, and KHJ. All authors contributed to the development of the protocol and manuscript and reviewed and approved the final version.

XSL•FO
RenderX

Conflicts of Interest

None declared.

References

1. Ghana Statistical Service (GSS), Ghana Health Service (GHS), ICF. Ghana Maternal Health Survey 2017: Key Findings. Rockville, MD: GSS, GHS, and ICF; 2018. URL: https://dhsprogram.com/pubs/pdf/SR251/SR251.pdf [accessed 2022-08-11]
2. U.S. Department of Health and Human Services. Healthy People 2010: Understanding and Improving Health. Washington, DC: U.S. Department of Health and Human Services, Government Printing Office; 2000. URL: https://files.eric.ed.gov/fulltext/ED443794.pdf [accessed 2022-08-11]
3. Ickovics J, Kershaw TS, Westdahl C, Magriples U, Massey Z, Reynolds H, et al. Group prenatal care and perinatal outcomes: A randomized controlled trial. Obstet Gynecol 2007 Aug;110(2 Pt 1):330-339 [FREE Full text] [doi: 10.1097/01.AOG.0000275284.24298.23] [Medline: 17666608]
4. Ickovics J, Earnshaw V, Lewis JB, Kershaw TS, Magriples U, Stasko E, et al. Cluster randomized controlled trial of group prenatal care: Perinatal outcomes among adolescents in New York City health centers. Am J Public Health 2016 Feb;106(2):359-365 [FREE Full text] [doi: 10.2105/AJPH.2015.302960] [Medline: 26691105]
5. Lori JR, Munro ML, Chuey MR. Use of a facilitated discussion model for antenatal care to improve communication. Int J Nurs Stud 2016 Feb;54:84-94 [FREE Full text] [doi: 10.1016/j.ijnurstu.2015.03.018] [Medline: 25862409]
6. Patil CL, Klima CS, Steffen AD, Leshabari SC, Pauls H, Norr KF. Implementation challenges and outcomes of a randomized controlled pilot study of a group prenatal care model in Malawi and Tanzania. Int J Gynaecol Obstet 2017 Dec;139(3):290-296 [FREE Full text] [doi: 10.1002/ijgo.12324] [Medline: 28905377]
7. McKinnon B, Sall M, Vandermorris A, Traoré M, Lamesse-Diedhiou F, McLaughlin K, et al. Feasibility and preliminary effectiveness of group antenatal care in Senegalese health posts: A pilot implementation trial. Health Policy Plan 2020 Jun 01;35(5):587-599. [doi: 10.1093/heapol/czz178] [Medline: 32155254]
8. Grenier L, Suhowatsky S, Kabue MM, Noguchi LM, Mohan D, Karnad SR, et al. Impact of group antenatal care (G-ANC) versus individual antenatal care (ANC) on quality of care, ANC attendance and facility-based delivery: A pragmatic cluster-randomized controlled trial in Kenya and Nigeria. PLoS One 2019;14(10):e0222177 [FREE Full text] [doi: 10.1371/journal.pone.0222177] [Medline: 31577797]
9. WHO Recommendations on Antenatal Care for a Positive Pregnancy Experience. Geneva, Switzerland: World Health Organization; 2016. URL: http://apps.who.int/iris/bitstream/10665/250796/1/9789241549912-eng.pdf?ua=1 [accessed 2021-07-01]
10. Lori JR, Ofosu-Darkwah H, Boyd CJ, Banerjee T, Adanu RMK. Improving health literacy through group antenatal care: A prospective cohort study. BMC Pregnancy Childbirth 2017 Jul 14;17(1):228 [FREE Full text] [doi: 10.1186/s12884-017-1414-5] [Medline: 28705179]
11. Squiers L, Peinado S, Berkman N, Boudewyns V, McCormack L. The health literacy skills framework. J Health Commun 2012;17 Suppl 3:30-54. [doi: 10.1080/10810730.2012.713442] [Medline: 23030560]
12. Ghana Statistical Service (GSS), Ghana Health Service (GHS), ICF International. Ghana Demographic and Health Survey 2014. Rockville, MD: GSS, GHS, and ICF International; 2015. URL: https://dhsprogram.com/pubs/pdf/FR307/FR307.pdf [accessed 2022-08-11]
13. Beck C, Lu B, Greevy R. Package ‘nbpMatching’: Functions for optimal non-bipartite matching. Version 1.5.1. The Comprehensive R Archive Network. 2016 Apr. URL: https://cran.r-project.org/web/packages/nbpMatching/nbpMatching.pdf [accessed 2022-08-11]
14. Greevy RA, Grijalva CG, Roumie CL, Beck C, Hung AM, Murff HJ, et al. Reweighted Mahalanobis distance matching for cluster-randomized trials with missing data. Pharmacoepidemiol Drug Saf 2012 May;21 Suppl 2:148-154 [FREE Full text] [doi: 10.1002/pds.3260] [Medline: 22552990]
15. Rotondi MA. Package ‘CRTSize’: Sample size estimation functions for cluster randomized trials. Version 1.0. Microsoft R Application Network. 2015 Apr. URL: https://mran.microsoft.com/snapshot/2016-09-10/web/packages/CRTSize/CRTSize.pdf [accessed 2022-08-14]
16. Kuganab-Lem RB, Dogudugu R, Kanton L. Birth preparedness and complication readiness: A study of postpartum women in a rural district of Ghana. Public Health Res 2014;4(6):225-233 [FREE Full text] [doi: 10.5923/j.phr.20140406.02]
17. Agresti A. Categorical Data Analysis. 3rd edition. Hoboken, NJ: John Wiley & Sons, Inc; 2013.
18. Holm S. A simple sequentially rejective multiple test procedure. Scand J Stat 1979;6(2):65-70 [FREE Full text]
19. Hsu JC. Multiple Comparisons: Theory and Methods. New York, NY: Chapman and Hall/CRC; 1996:1-296.
20. Bates D, Mächler M, Bolker B, Walker S. Fitting linear mixed-effects models using lme4. J Stat Softw 2015;67(1):1-48 [FREE Full text] [doi: 10.18637/jss.v067.i01]
21. Campbell MK, Piaggio G, Elbourne DR, Altman DG, CONSORT Group. CONSORT 2010 statement: Extension to cluster randomised trials. BMJ 2012 Sep 04;345:e5661. [doi: 10.1136/bmj.e5661] [Medline: 22951546]
22. Steckler A, Linnan L, editors. Process Evaluation for Public Health Interventions and Research. San Francisco, CA: Jossey-Bass; 2002.

XSL•FO
RenderX

23. Harris PA, Taylor R, Thielke R, Payne J, Gonzalez N, Conde JG. Research electronic data capture (REDCap)--A metadata-driven methodology and workflow process for providing translational research informatics support. J Biomed Inform 2009 Apr;42(2):377-381 [FREE Full text] [doi: 10.1016/j.jbi.2008.08.010] [Medline: 18929686]
24. Harris PA, Taylor R, Minor BL, Elliott V, Fernandez M, O'Neal L, REDCap Consortium. The REDCap Consortium: Building an international community of software platform partners. J Biomed Inform 2019 Jul;95:103208 [FREE Full text] [doi: 10.1016/j.jbi.2019.103208] [Medline: 31078660]
25. Ending Preventable Maternal Mortality: USAID Maternal Health Vision for Action Evidence for Strategic Approaches. Washington, DC: U.S. Agency for International Development; 2015 Jan. URL: http://www.usaid.gov/sites/default/files/documents/1864/MH%20Strategy_web_red.pdf [accessed 2021-06-22]
26. Nutbeam D. Health literacy as a public health goal: A challenge for contemporary health education and communication strategies into the 21st century. Health Promot Int 2000 Sep;15(3):259-267 [FREE Full text] [doi: 10.1093/heapro/15.3.259]
27. Sørensen K, Van den Broucke S, Fullam J, Doyle G, Pelikan J, Slonska Z, (HLS-EU) Consortium Health Literacy Project European. Health literacy and public health: A systematic review and integration of definitions and models. BMC Public Health 2012 Jan 25;12:80 [FREE Full text] [doi: 10.1186/1471-2458-12-80] [Medline: 22276600]
28. von Wagner C, Steptoe A, Wolf M, Wardle J. Health literacy and health actions: A review and a framework from health psychology. Health Educ Behav 2009 Oct;36(5):860-877. [doi: 10.1177/1090198108322819] [Medline: 18728119]
29. Renkert S, Nutbeam D. Opportunities to improve maternal health literacy through antenatal education: An exploratory study. Health Promot Int 2001 Dec;16(4):381-388. [doi: 10.1093/heapro/16.4.381] [Medline: 11733456]
30. Batterham R, Hawkins M, Collins P, Buchbinder R, Osborne R. Health literacy: Applying current concepts to improve health services and reduce health inequalities. Public Health 2016 Mar;132:3-12. [doi: 10.1016/j.puhe.2016.01.001] [Medline: 26872738]

Abbreviations

ANC: antenatal care
CONSORT: Consolidated Standards of Reporting Trials
GRAND: Group Antenatal Care Delivery Project
ICC: intraclass correlation coefficient
IRB: Institutional Review Board
NIH: National Institutes of Health
RA: research assistant
RCT: randomized controlled trial
REDCap: research electronic data capture
WHO: World Health Organization

Edited by T Leung;y the Community-Level Health Promotion (CLHP) Study Section - Healthcare Delivery and Methodologies Integrated Review Group - Center for Scientific Review (National Institutes of Health, USA). Submitted 14.07.22; accepted 30.07.22; published 09.09.22.

Please cite as:
Lori JR, Williams JEO, Kukula VA, Apetorgbor VEA, Awini EA, Amankwah G, Zielinski R, Lockhart N, James KH, Moyer CA
Group Antenatal Care in Ghana: Protocol for a Cluster Randomized Controlled Trial
JMIR Res Protoc 2022;11(9):e40828
URL: https://www.researchprotocols.org/2022/9/e40828
doi: 10.2196/40828
PMID:

©Jody R Lori, John E O Williams, Vida A Kukula, Veronica E A Apetorgbor, Elizabeth A Awini, Georgina Amankwah, Ruth Zielinski, Nancy Lockhart, Katherine H James, Cheryl A Moyer. Originally published in JMIR Research Protocols (https://www.researchprotocols.org), 09.09.2022. This is an open-access article distributed under the terms of the Creative Commons Attribution License (https://creativecommons.org/licenses/by/4.0/), which permits unrestricted use, distribution, and reproduction in any medium, provided the original work, first published in JMIR Research Protocols, is properly cited. The complete bibliographic information, a link to the original publication on https://www.researchprotocols.org, as well as this copyright and license information must be included.

XSL•FO
RenderX
